# Blueberry Component Pterostilbene Protects Corneal Epithelial Cells from Inflammation via Anti-oxidative Pathway

**DOI:** 10.1038/srep19408

**Published:** 2016-01-14

**Authors:** Jin Li, Ruzhi Deng, Xia Hua, Lili Zhang, Fan Lu, Terry G. Coursey, Stephen C. Pflugfelder, De-Quan Li

**Affiliations:** 1 Zhejiang Eye Hospital, School of Optometry and Ophthalmology, Wenzhou Medical University, Wenzhou, China; 2 Ocular Surface Center, Cullen Eye Institute, Department of Ophthalmology, Baylor College of Medicine, Houston, Texas, USA; 3 Tianjin Eye Hospital, Tianjin Key Lab of Ophthalmology and Visual Science, Clinical College of Ophthalmology, Tianjin Medical University, Tianjin, China

## Abstract

Blueberries have been recognized to possess protective properties from inflammation and various diseases, but not for eye and ocular disorders. This study explores potential benefits of pterostilbene (PS), a natural component of blueberries, in preventing ocular surface inflammation using an *in vitro* culture model of human corneal epithelial cells (HCECs) exposed to hyperosmotic medium at 450 mOsM. Gene expression was detected by RT-qPCR, and protein production or activity was determined by ELISA, zymography, Western blotting and immunofluorescent staining. Reactive oxygen species (ROS) production was measured using DCFDA kit. The addition of PS significantly reduced the expression of pro-inflammatory mediators, TNF-α, IL-1 β, IL-6, MMP-2 and MMP-9 in HCECs exposed to hyperosmotic medium. Pre-treatment with PS (5 to 20 μM) suppressed ROS overproduction in a dose-dependent manner. Additionally, PS significantly decreased the levels of oxidative damage biomarkers, malondialdehyde (MDA), 4-hydroxynonenal (4-HNE), aconitase-2 and 8-hydroxydeoxyguanosine (8-OHdG). Importantly, PS was found to rebalance homeostasis between oxygenases and anti-oxidative enzymes by decreasing cyclooxygenase 2 (COX2) expression and restoring the activity of antioxidant enzymes, superoxide dismutase 1 (SOD1) and peroxiredoxin-4 (PRDX4) during hyperosmotic stress. Our findings demonstrate that PS protects human cornea from hyperosmolarity-induced inflammation and oxidative stress, suggesting protective effects of PS on dry eye.

Blueberry consumption has been known to benefit health with preventive effects on cardiovascular disease, neurodegeneration, diabetes, cancer and inflammation. Various animal studies and clinical trials suggest eating blueberries may lower the risk of myocardial infarction and breast cancer, decrease blood pressure, improve insulin resistance and endothelial function, as well as reduce the inflammation^1^,^2^,^3^,^4^. These protective effects can mostly be attributed to blueberry natural component pterostilbene (PS), a phytoalexin that protects plants from inflammatory injures. PS, an analog of resveratrol, is lipophilic and oral-soluble with 20–80% higher bioavailability than resveratrol, making it an attractive potential therapeutic agent (see review articles^5^,^6^).

The effect of PS centers around its suppressive effects on inflammation, apoptosis and oxidative stress^7^. PS has been reported to suppress the production and signaling pathways of proinflammatory cytokines (TNF-α, IL-1 β, IL-4), matrix metalloproteinases (MMPs), cyclooxygenase (COX) 2, MAP kinases and NF-kB p65 phosphorylation^8^,^9^. PS protects vascular endothelial cells against oxidized low-density lipoprotein-induced apoptosis through a pathway involving oxidative stress, p53, mitochondria, cytochrome C and caspase protease^10^. PS has been shown to suppress breast cancer stem cells with reducing the stem cell surface antigen CD44 and promoting beta-catenin phosphorylation through inhibition of hedgehog/Akt/GSK3 β signaling and downstream molecules, c-myc and cyclin D1^11^. PS is also a potent neuromodulator for aging and Alzheimer’s disease^12^. However, little is known about the potential benefits and therapeutic potential of blueberries and their natural compounds in eye and ocular surface diseases.

Dry eye disease is a multifactorial disease of tear and ocular surface that results in symptoms of discomfort, visual disturbance and tear instability with potential damage to the ocular surface^13^. It is often accompanied by increased osmolarity of the tear film and inflammation of the ocular surface^14^,^15^,^16^. Dry eye disease affects the lives of millions of people, as the prevalence is as high as 14.5 percent (17.9 percent in women and 10.5 percent in men) and continues to rise^17^,^18^. An important mechanism of dry eye pathogenesis is hyperosmolarity due to deficient tear production and/or tear over evaporation. This causes tear film instability, impaired mucin expression, ocular surface inflammation, corneal epithelial apoptosis, and goblet cell loss^17^.

Studies, both *in vivo* and *in vitro*, have demonstrated that reactive oxygen species (ROS) overproduction and oxidative stress are an underlying mechanism of many ocular surface diseases including dry eye^19^,^20^,^21^. Thus, molecules that suppress inflammation and oxidative damage would be strong potential candidates to prevent or treat eye diseases in which oxidative stress-induced inflammation plays a role in disease pathogenesis. A variety of dry eye treatments, such as tear supplementation, has focused on reducing tear hyperosmolarity. Osmoprotectants and antioxidants, such as L-carnitine, have been shown to protect ocular surface from inflammation and oxidative injury in human corneal epithelial cells and murine dry eye models^22^,^23^,^24^,^25^. We hypothesize that PS, as a natural dietary component of blueberries, may have potential benefits to dry eye disease based on its protective effect against inflammation and oxidative stress in other organs and tissues. The present study explores the protective role and underlining mechanisms of PS in prevention of inflammatory injury in primary human corneal epithelial cells (HCECs) under hyperosmotic stress, an *in vitro* dry eye model.

## Results

### PS significantly decreased the expression of pro-inflammatory mediators in HCECs exposed to hyperosmotic stress

**Figure 1:**
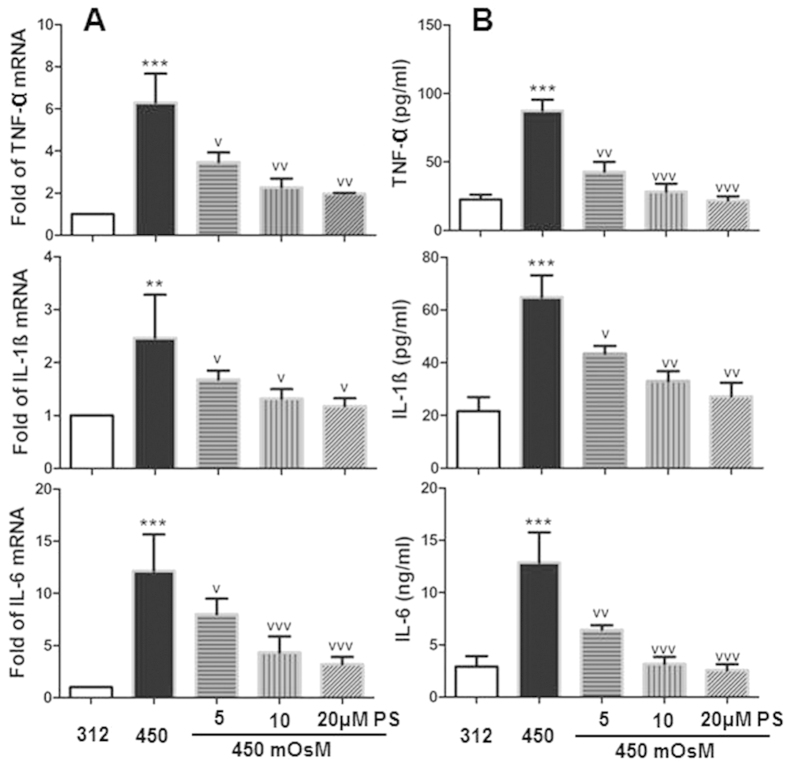
Pterostilbene (PS) suppressed expression of proinflammatory cytokines TNF-α, IL-1 β and IL-6 in HCECs exposed to hyperosmotic medium. Primary HCECs were cultured in isomolar (312 mOsm) medium, then switched to hyperosmotic medium (450 mOsm) alone or in the presence of PS in different concentrations (5, 10 and 20 μM) for 4 h to evaluate mRNA level by RT-qPCR (**A**); or for 24 h to measure protein level by ELISA (**B**). Data were summarized as mean ± SD from 5 separated experiments. **P < 0.01, ***P < *0.001,* as compared with 312 mOsM; ^v^*P* < *0.05,*^vv^*P* < *0.01,*^vvv^*P* < 0.001, as compared with 450 mOsM.

Our previous studies demonstrated that hyperosmolarity significantly increased the expression of pro-inflammatory cytokines, chemokines^26^ and MMPs^27^, and the present study further confirms these findings. As shown in Fig. 1A, treatment with medium at 450 mOsm increased mRNA expression of TNF-α, IL-1 β and IL-6 to 6.30 ± 1.37, 2.47 ± 0.81 and 12.15 ± 3.49 fold (P < 0.001, 0.01, and 0.001, respectively) compared with normal control (312 mOsM). The expression of these three cytokines decreased to 3.47 ± 0.46 (P < 0.05), 1.68 ± 0.16 (P < 0.05), and 8.01 ± 1.48 fold (P < 0.05), respectively, in HCECs at 450 mOsM with addition of 5 μM PS. These cytokines further decreased to 2.28 ± 0.40, 1.32 ± 0.18 and 4.35 ± 1.53 fold (P < 0.01, 0.05, 0.001) by 10 μM PS, and 1.97 ± 0.04, 1.18 ± 0.15 and 3.21 ± 0.69 fold (P < 0.01, 0.05, 0.001), respectively, by 20 μM PS. Evaluated by ELISA, protein production of TNF-α, IL-1 β and IL-6 were 22.53 ± 3.62 pg/ml, 21.66 ± 5.32 pg/ml and 2.93 ± 0.99 ng/ml in HCECs at isomolar condition. Hyperosmotic medium (450 mOsM) increased their production to 87.57 ± 7.96 pg/ml, 64.92 ± 8.22 pg/ml and 12.90 ± 2.86 ng/ml, respectively. Interestingly, prior treatment with 5, 10 and 20 μM of PS significantly reduced production of these pro-inflammatory cytokines to 42.96-21.89 pg/ml, 43.41-27.21 pg/ml and 6.45-2.57 ng/ml, respectively in a concentration-dependent manner. These results suggest that PS has a suppressive effect on inflammatory biomarkers at both mRNA and protein levels.

**Figure 2:**
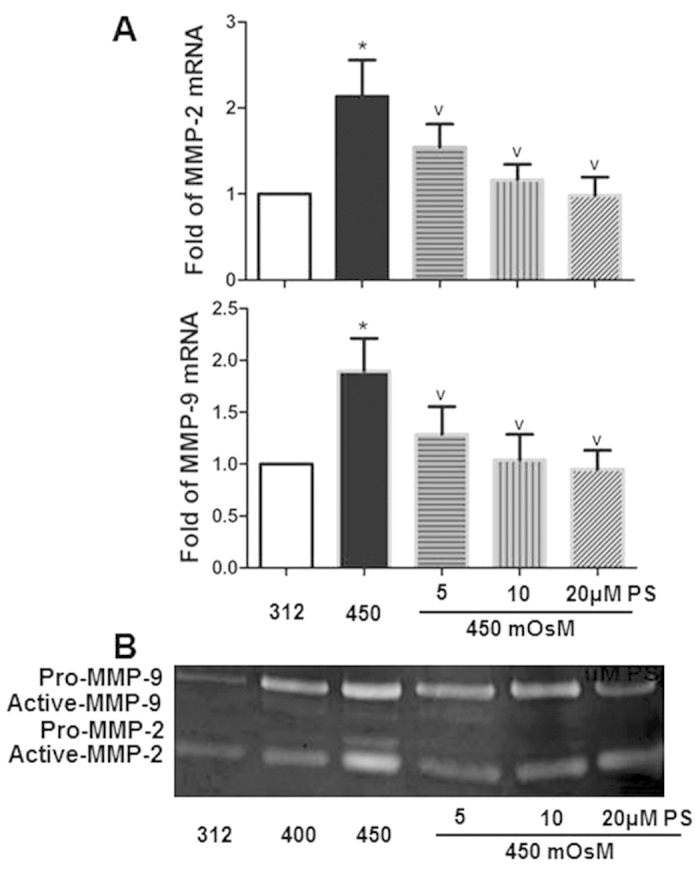
PS down-regulated expression of MMP-2 and -9 in HCECs exposed to hyperosmotic medium. Primary HCECs were cultured in isomolar (312 mOsm) medium, then switched to hyperosmotic medium (450 mOsm) alone or in the presence of PS in different concentrations (5, 10 and 20 μM) for 4 h to evaluate mRNA level by RT-qPCR (**A**); or for 24 h to determine protein production and activity of MMP-2 and -9 by zymography (**B**). Data were summarized or representative from 3 separated experiments. *P < *0.05,* as compared with 312 mOsM; ^v^*P* < *0.05,* as compared with 450 mOsM.

Hyperosmolarity significantly increased the expression of MMP-2 and MMP-9. Compared to the normal isomolar control, mRNA expression increased to 2.13 ± 0.42 for MMP-2 and 1.90 ± 0.31 fold for MMP-9 (all P < 0.05) in HCECs exposed to hyperosmotic medium (450 mOsM). Treatment with PS at 5, 10 or 20 μM significantly reduced MMP-2 and MMP-9 transcripts (all P < 0.05, Fig. 2A). Gelatin zymography confirmed that protein production and activity of MMP-2 and MMP-9 had a similar pattern as mRNA levels.

### Treatment with PS significantly reduced hyperosmolarity-induced ROS overproduction

**Figure 3:**
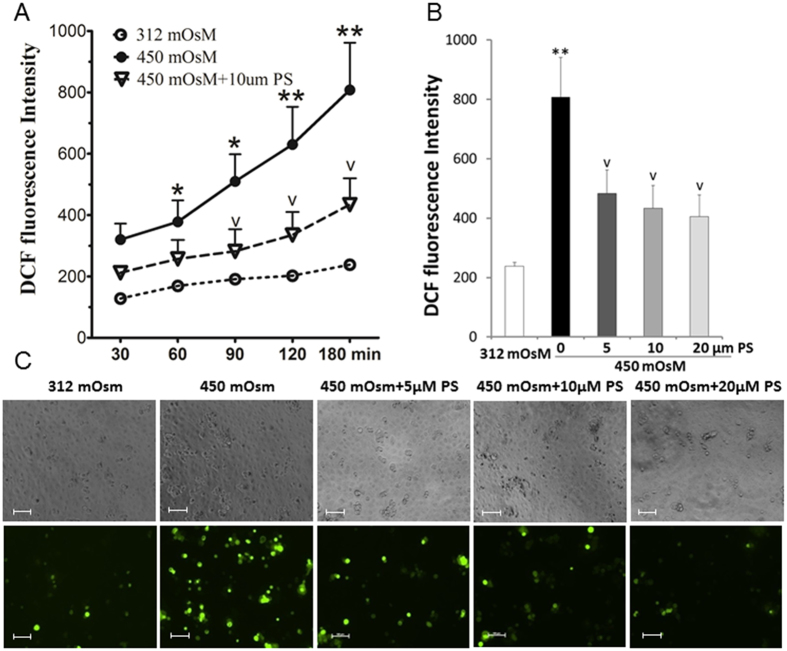
PS blocked overproduction of reactive oxygen species (ROS) in HCECs exposed to hyperosmotic medium. Primary HCECs were cultured in isomolar (312 mOsm) medium, then switched to hyperosmotic medium (450 mOsm) alone or in the presence of PS in different concentrations (5, 10 and 20 μM) for 3 h. ROS was analyzed with 2′, 7′-dichlorofluorescein diacetate (DCFDA) kit. (**A**) ROS production by HCECs in medium at 312 or 450 mOsM without or with 10 μM PS during 30–180 min, as measured by DCF fluorescence intensity. (**B**) ROS production suppressed by PS at different concentrations (5, 10, 20 μM) at 180 min. (**C**) The representative images showed positive cells of ROS-DCF fluorescence in HCECs at 450 mOsM for 120 min without or with 5–20 μM PS; the phase images showed equal cell density in 5 groups. Magnification 100x. Data were summarized or representative from 3 separated experiments. *P < 0.05, **P < *0.01*, as compared with 312 mOsM; ^v^*P* < *0.05*, as compared with 450 mOsM.

We next sought to determine the underlining mechanism by which PS protects HCECs from inflammatory injury induced by hyperosmolarity. To accomplish this we used a DCFDA (2′,7′-dichlorofluorescein diacetate) assay to detect all forms of ROS generated during cell metabolism, including superoxide ion (O_2_^−^), hydroxy radicle (HO•) and hydrogen peroxide (H_2_O_2_). Both DCF fluorescence intensity measurement and microscopy observation revealed that hyperosmotic stress stimulated intracellular ROS production. By contrast, PS pretreatment significantly reduced ROS levels. As shown in Fig. 3A, a time-course study (30–180 min) of DCF fluorescence intensities suggests that ROS generation increases in a time-dependent manner after exposure to 450 mOsM. However, ROS levels remain at low relatively stable levels at normal isomolar condition. PS (10 μM) treatment reduced intensity from 780 ± 110 to 410 ± 65 at 180 min. Overall, ROS generation was suppressed with 5 to 20 μM PS in a concentration- dependent manner (Fig. 3B). DCF fluorescence microscopy further showed that ROS fluorescence-positive cells increased dramatically from 8.3% in isomolar control to 28% in cells at 450 mOsM. It was reduced significantly to 13.7, 9.8 and 7.2% with prior incubated PS at 5, 10 and 20 μM (Fig. 3C).

### PS reduced oxidative damage induced by hyperosmotic stress in HCECs

**Figure 4:**
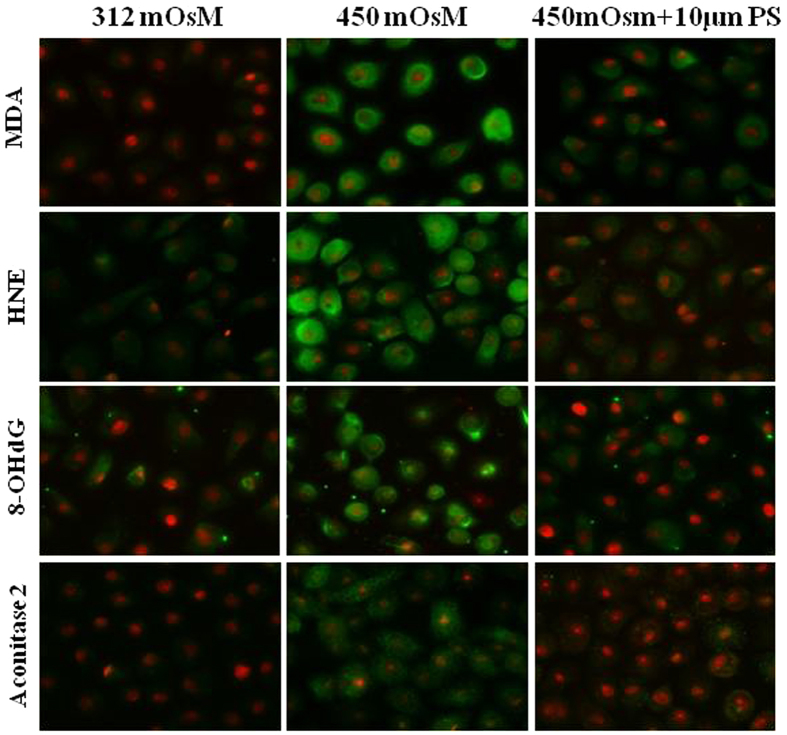
Representative images showed that PS reduced the oxidative damage. Immunofluorescent staining showed the biomarkers of lipid peroxidation, MDA and HNE, and mitochondrial DNA damage, 8-OHdG and aconitase 2, in primary HCECs exposed to 450 mOsM medium without or with 10 μM PS for 24 h. Data were representative of 3 separate experiments.

ROS overproduction can induce cell oxidative damage. To evaluate oxidative stress we examined the production of lipid peroxidation biomarkers malondialdehyde (MDA) and 4-hydroxy-2-nonenal (HNE). To examine mitochondrial DNA damage, the levels of biomarkers 8-hydroxy-2-deoxyguanosine (8-OHdG) and aconitase-2 were determined. As shown in Fig. 4, immunofluorescent staining demonstrates that expressions of these biomarkers was very low under normal isomolar conditions. However, all four biomarkers increased with exposure to hyperosmotic medium for 24 hours. The positive staining cells for each biomarker increased from 7.4% to 81.4% (MDA), 20.0% to 84.0% (4-HNE), 16.7% to 91.7% (8-OHdG) and 7.6% to 92.3% (aconitase-2). Prior incubation of 10 μM PS significantly reduced their levels to 29.6% (MDA), 24.0% (4-HNE), 29.2% (8-OHdG) and 19.2% (aconitase-2), respectively.

### PS restored the balance of oxygenases and antioxidative enzymes

**Figure 5:**
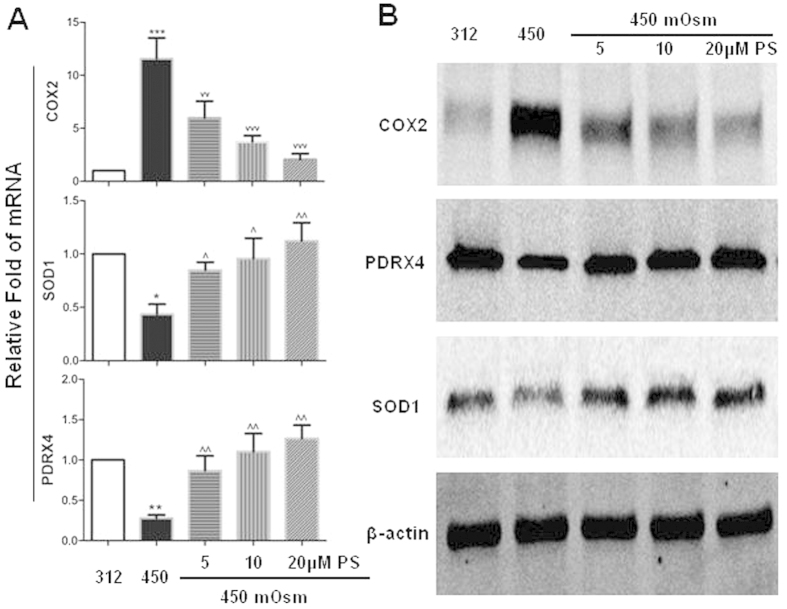
Pterostilbene (PS) rebalances the expression between oxygenase COX2 and antioxidative enzymes SOD1 and PRDX4 in HCECs exposed to 450 mOsM. (**A**) mRNA expression evaluated by RT-qPCR; (**B**) protein levels by Western blot. Data were summarized from 3 separated experiments. *P < 0.05, **P < 0.01, ***P < *0.001*, as compared with 312 mOsM; ^vv^*P* < *0.01,*^vvv^*P* < *0.001*, ^*P* < *0.05*, ^^*P* < *0.01*, as compared with 450 mOsM.

We evaluated the mRNA and protein expression levels of oxidative stress-associated enzymes. COX2, officially as prostaglandin-endoperoxide synthase, is an enzyme that mediates oxidative stress^28^. Superoxide dismutase-1 (SOD1) and peroxiredoxin-4 (PRDX4), both antioxidant enzymes, significantly declined during increased levels of oxidative stress^29^,^30^. As shown in Fig. 5A, the mRNA level of COX2 increased markedly to 11.57 ± 1.95 fold (P < 0.001) in HCECs exposed to hyperosmotic medium (450 mOsM) compared with isomolar control. However, it was significantly reduced to 5.98 ± 1.58, 3.72 ± 0.59 and 2.05 ± 0.56 fold, respectively, when treated with PS at increasing concentrations (5, 10 and 20 μM). By contrast, the mRNA expression of SOD1 and PDRX4 significantly decreased to 0.43 ± 0.09 and 0.28 ± 0.04, respectively, in HCECs exposure to hyperosmolarity. Interestingly, their mRNA levels rebound significantly in response to PS treatment with increasing concentrations (5, 10 and 20 μM).

Western blot analysis confirmed the increase of COX2 and decrease of SOD1 and PDRX4 at protein levels in HCECs exposed to 450 mOsM (Fig. 5B). The results were consistent with our previous report^20^ that revealed an imbalance between oxygenases and antioxidant enzymes in HCECs under hyperosmotic stress. This could be an important mechanism causing excessive ROS production and oxidative damage in HCECs. Interestingly, PS largely suppressed COX2 production while increased the SOD1 and PDRX4 to near normal levels at protein levels (Fig. 5B), restoring the balance of oxygenases and antioxidative enzymes in response to hyperosmotic stress.

## Discussion

The protective properties of the dietary components of blueberries have been recognized to suppress inflammation, reduce risk of carcinogenesis, ameliorate diabetes, and attenuate vascular and neurological diseases^2^,^7^. However, little is known about the bioactivity and potential clinical implications of blueberry components on the health of the human eye. Here, we present evidence that PS may potentially have beneficial effects on eye and ocular surface diseases. We show that PS suppresses the expression of inflammatory mediators, reduces ROS generation, and attenuates oxidative damage in cellular membrane, nuclear and mitochondria through rebalancing activity between oxygenases and antioxidative enzymes in HCECs exposed to hyperosmotic stress.

Many studies, including our own, have reported that hyperosmotic stress can elicit an inflammatory response through different proinflammatory mediators, such as TNF-α, IL-1 β, IL-6, MMP-2, -9 and -3. An increase in these molecules has been found in the HCEC culture model, the *in vivo* murine dry eye model, and in the tear fluid of dry eye patients^31^,^32^,^33^. This study confirms previous findings and further revealed that PS could successfully suppress the proinflammatory response when HCECs exposed to hyperosmotic stress.

Multiple signal pathways have been reported to explain the activity of PS. For example, dietary intake of PS inhibits p38-induced inflammatory markers and nuclear phospho-p65 in colon cancer^8^,^34^. PS also attenuates inflammation in rat heart due to cardiac ischemia through TLR4/NF-κB signaling pathway^35^. Many other reports show that PS plays an important role in reducing inflammation in cancer, ischemia, cognition decline and diabetes^6^. Here our findings demonstrate for the first time that PS suppresses production of proinflammatory biomarkers TNF-α, IL-1 β, IL-6, MMP-2 and -9 in HCECs exposed to hyperosmotic stress, providing the evidence that PS is a protector of corneal epithelium from inflammatory injures.

Accumulating evidence suggests that oxidative stress played an important role in ocular surface diseases, including dry eye^19^,^20^,^36^. *In vivo* lipid oxidative injury has been observed in dry eye and Sjögren syndrome patients^21^. Higher oxidative stress may also explain the higher incidence and more severe dry eye in elder patients^37^. *In vitro* studies further revealed that lipid and mitochondrial oxidative damage may cause inflammation in HCECs^38^. However, the role and mechanism of oxidative stress in inflammation remain elusive.

PS is a known antioxidant molecules with anti-inflammatory effects^8^. To date, little is known about protective effect of PS on eye and ocular surface diseases. Here, we performed comprehensive studies to explore the beneficial effects of PS using an *in vitro* HCEC culture model that mimics dry eye that occurs under hyperosmotic stress. Our findings demonstrate that hyperosmotic stress induces inflammation and oxidative injuries and PS is an effective agent that protects HCECs from hyperosmolarity-induced inflammation.

The present study further showed that PS suppresses hyperosmolarity-induced inflammation via its protective effects against oxidative stress. PS was observed to protect HCECs from oxidative stress by suppressing ROS overproduction and reducing ROS-induced cellular oxidative damage. Lipid peroxidation of the corneal epithelium causes changes in the fluidity and permeability of cell membranes and impairs the activity of membrane-bound enzymes. MDA and 4-HNE are two important end-products of oxidation of polyunsaturated fatty acids, and frequently measured as indicators of lipid peroxidation and oxidative stress^39^. Aconitase activity and 8-OHdG levels are major markers for the mitochondria DNA damage^40^,^41^. Aconitase-2 is an iron-sulfur protein which acts as sensor in the redox regulation of metabolism by O_2_. Aconitase-2 increases with high mitochondrial activity in the cells under oxidative stress. 8-OHdG is a product of oxidative DNA damage following specific enzymatic cleavage in mitochondrial and nuclear DNA. The released 8-OHdG has been widely used as a sensitive and reliable marker of the oxidative DNA damage. Our data showed that PS reduces the lipid peroxidation markers MDA and 4-HNE, as well as mitochondrial DNA damage markers 8-OHdG and aconitase 2 in HCECs exposed to hyperosmotic stress.

Furthermore, PS was found to modulate the oxygenase and antioxidative enzymes associated to oxidative stress. COX2 is considered a pro-inflammatory enzyme as free radicals and prostaglandins are produced during its catalytic cycle. Upregulation of COX2 is a common feature of inflammation by oxidative stress^42^,^43^. As major antioxidative enzymes, SOD1 plays a crucial role in scavenging O_2_^− ^44. PRDX4, as a guardian against oxidative stress, also modulate oxidative signaling^30^. These enzymes, often present at high levels and capable of rapidly clearing peroxides, display a remarkable array of variations in their oligomeric states and susceptibility to regulation by hyperoxidative inactivation and other post-translational modifications^45^,^46^. PS was found to inhibit pancreatic cancer growth *in vivo*, which was associated with increasing expression of antioxidative enzyme SOD and anti-proliferation function^47^. Previous studies also suggest that induction of antioxidant enzymes by pterostilbene could protect from cardiovascular disease^48^. The use of PS could reduce the oxidative damage from smoking, alcohol, and gastroesophageal reflux disease. Our data further demonstrate that PS protects HCECs from oxidative damage by reducing oxygenase COX2 levels while increasing the production of anti-oxidative enzymes SOD1 and PRDX4, restoring the balance between oxygenases and antioxidative enzymes.

These findings suggest that a shift of strategies for dry eye treatment from simple tear-related interventions to a combination with agents that protect eye from inflammatory injures. Corticosteroids, cyclosporin and doxycycline are used to treat inflammation in dry eye. However, all these agents have significant side effects, such as infection susceptibility and tissue necrosis, often limiting their use. The development of a natural dietary component for dry eye prevention and treatment would be a novel strategy that would benefit a large population patients suffering with eye disorders.

In summary, the present study further reveals a novel phenomenon that a natural blueberry component, PS, effectively protects human corneal epithelium from hyperosmolarity-induced inflammation by reducing ROS generation and oxidative damage through rebalancing the production and activity between oxygenases and antioxidative enzymes. Our findings for the first time suggest potential benefits of blueberries and their natural components on ocular surface disorders, such as dry eye.

## Materials and Methods

### Materials and Reagents

Pterostilbene was purchased from Sigma-Aldrich (St. Louis, MO). Cell culture supplies such as Dulbecco modified Eagle medium (DMEM), Ham-F12, Cortisone, EGF, gentamicin, amphotericin B were obtained from Invitrogen (Grand Island, NY); Fetal bovine serum (FBS) from Hyclone (Logan, UT). DCFDA-cellular ROS detection assay and rabbit polyclonal antibodies against MDA, 4-HNE, aconitase-2 or 8-OHdG were from Abcam (Cambridge, MA). Fluorescein Alexa-Flour 488-conjugated secondary antibodies (goat anti rabbit or mouse IgG, rabbit or donkey anti goat IgG) were from Molecular Probes (Eugene, OR). RNeasy Plus Mini RNA extraction kit from Qiagen (Valencia, CA). TaqMan gene expression assays and real-time PCR master mix from Applied Biosystems (Foster City, CA). Ready-To-Go You-Prime First-Strand Beads were from GE Healthcare (Piscataway, NJ). ELISA kits for TNF-α, IL-1 β and IL-6 were from Biolegend (San Diego, CA). Gelatin zymogram gels were from Bio Rad (Hercules, CA). All plastic ware were purchased from Becton Dickinson Biosciences (Lincoln Park, NJ).

### Cultures of primary HCECs and *in vitro* model of hyperosmotic stress

Corneas from donors (19–67 years old) within 72 h after death were obtained from Lions Eye Bank of Texas (Houston, TX). Primary HCECs were cultured from limbal explants in a supplemented hormonal epidermal medium (SHEM) containing 5% FBS according to our previous method^49^. Hyperosmotic stress model was established by switching HCECs from isosmotic (312 mOsM) to hyperosmotic medium at 400 and 450 mOsM, which was achieved by adding 49 or 69 mM sodium chloride^16^,^50^. To study its effects, different concentrations (5, 10, 20 μM) of PS were co-incubated in certain wells. The cells treated for 4 hours were used for RNA extraction. The cells treated for 24 hours were used for immunostaining, ELISA, or lysed in RIPA buffer for Western blot analysis.

### RNA extraction, reverse transcription, and quantitative real-time PCR (RT-qPCR)

Total RNA was extracted with RNeasy Plus Mini Kit (Qiagen, Valencia, CA) according to the manufacturer’s instructions, quantified with a spectrophotometer (NanoDrop ND-1000; Thermo Scientific, Wilmington, DE), and stored at −80 °C before use. The first strand cDNA was synthesized by RT from 2.0 μg of total RNA using Ready-To-Go You-Prime First-Strand Beads as previously described^51^. Quantitative real-time PCR was performed in StepOnePlus™ Real-Time PCR System (Applied Biosystems, Foster City, CA) with 10 μl reaction volume containing 4 μl of cDNA, 0.3 μl TaqMan gene expression assay, 5 μl TaqMan gene expression master mix and 0.7 μl H_2_O. TaqMan gene expression assays used for this study were: GAPDH (Hs99999905_m1), TNF-α (Hs00174128_m1), IL-1 β (Hs01555413_m1), IL-6 (Hs00174131_m1), MMP-2 (Hs01548724_m1), MMP-9 (Hs00234579_m1), COX2 (Hs00153133_m1), SOD1 (Hs00533490_m1), PRDX 4 (Hs01056076_m1). The thermocycler parameters were 50 °C for 2 min and 95 °C for 10 min, followed by 40 cycles of 95 °C for 15 s and 60 °C for 1 min. A non-template control was included to evaluate DNA contamination. The results were analyzed by the comparative threshold cycle (Ct) method and normalized by GAPDH as an internal control^52^.

### Enzyme-linked immunosorbent assays

Double-sandwich ELISA for human TNF-α, IL-1 β, IL-6 was performed to determine the protein concentration of these pro-inflammatory cytokines in the conditioned media from different treatments according to our previously reported protocol^16^. Absorbance was read at 450 nm with a reference wavelength of 570 nm by Infinite M200 microplate reader (Tecan US, Inc., Morrisville, NC).

### Gelatin zymography

Sodium dodecyl sulfate–polyacrylamide gel electrophoresis (SDS–PAGE) gelatin zymography was performed referring to a previously reported method^53^. Briefly, 10 μl of each conditioned medium was treated with SDS sample buffer without boiling. Samples were fractionated in a 10% polyacrylamide gelatin gel with electrophoresis at 100 V for 90 min at 4 °C. The gels were soaked in 0.25% Triton X-100 for 30 min at room temperature to remove the SDS, and incubated in a digestion buffer (50 mM Tris-HCl, pH 7.4, 150 mM NaCl, 10 mM CaCl2, 2 μM ZnSO4, and 0.01% Brij-35) containing 5 mM phenylmethylsulfonyl fluoride (PMSF), a serine protease inhibitor, at 37 °C overnight to allow proteinase digestion of its substrate. Gels were rinsed again in distilled water, stained with 0.25% Coomassie brilliant blue R-250 in 40% isopropanol for 2 h, and destained with 7% acetic acid. Gelatinolytic activities appeared as clear bands of digested gelatin against a dark blue background of stained gelatin.

### Measurement of cellular ROS production

Cellular ROS production was measured using DCFDA assay kit as we previously reported^20^. DCFDA, a cell-permeable fluorogenic dye, is deacetylated by cellular esterases to a non-fluorescent compound, it could be oxidized by ROS into highly fluorescent 2′,7′-dichlorofluorescein (DCF) which measures hydroxyl, peroxyl and other ROS activity within the cell. HCECs were grown on the 96-well plates or 8-chamber slides. When confluent, HCECs were incubated with 25 μM DCFDA at 37 °C for 45 min, and then exposed to hyperosmotic medium (450 mOsM) alone or in the presence of 10 μM of PS for different time periods (30–180 min). Cell images were taken with fluorescence microscope. Cell fluorescence in 96-well plates was measured at 488 nm excitation and 525 nm emission using Tecan Infinite M200 Microplate Reader (Tecan US, Inc. Morrisville, NC). Relative changes of DCF fluorescence were also expressed as fold increase over the control cells at isosmolar condition.

### Immunofluorescent Staining

Using a previously reported method^54^, HCECs on 8-chamber slides were fixed with 4% paraformaldehyde for 10 min and then permeabilized with 0.2% Triton X-100 in PBS at room temperature for 10 min. The cells were then incubated with primary antibodies against human MDA, HNE, aconitase-2 or 8-OHdG at 4 °C overnight. Alexa-Fluor 488 conjugated secondary antibodies was applied, and propidium iodide (PI) was used for nuclear counterstaining. The stained slides were photographed with Zeiss laser scanning confocal microscope (LSCM510META, Thornwood, NY).

### Western Blot Analysis

Western blot analysis was performed as our previous report^16^. Equal amounts of protein measured by BCA protein assay kit were mixed with 6 × SDS reducing sample buffer and boiled for 5 minutes before loading. The proteins (50 μg/lane) were separated on an SDS polyacrylamide gel and transferred electronically to PVDF membranes. After blocked with 5% nonfat milk in TTBS (50 mM Tris [pH 7.5], 0.9% NaCl, and 0.1% Tween-20) for 1 h, the membranes were incubated with primary antibodies against COX2 (1:200), SOD1 (1:200), PDRX4 (1:200) or β-actin (1:1000) at 4 °C overnight, then incubated with HRP conjugated goat anti-mouse or goat anti-rabbit IgG (1:1000) for 1 h. The signals of the bands were detected with a chemiluminescence reagent (ECL, GE Healthcare) using Kodak 4000R imaging station (Eastman Kodak, Rochester, NY).

### Statistical analysis

Student’s t-test was used to compare differences between two groups. One-way ANOVA test was used to make comparisons among three or more groups, followed by Dunnett’s post-hoc test. P  < 0.05 was considered statistically significant.

## Additional Information

**How to cite this article**: Li, J. *et al*. Blueberry Component Pterostilbene Protects Corneal Epithelial Cells from Inflammation via Anti-oxidative Pathway. *Sci. Rep.* **6**, 19408; doi: 10.1038/srep19408 (2016).
